# The Prevalence and Associated Risk Factors for Neonatal Thrombocytopenia Among Newborns Admitted to the Neonatal Intensive Care Unit

**DOI:** 10.7759/cureus.56108

**Published:** 2024-03-13

**Authors:** Hussain A Al Ghadeer, Rahmah A Aldhahi, Faisal K Al Dandan, Mohammed H Alamer, Luay F Almulaifi, Abdullah F Al Muaibid, Qesmah A Al-Ali, Tawfiq M Aljubran, Abdullah A Alarbash, Zahra E Alabbad, Amal S Alsultan, Zahra H Aldoukhi, Amjad A Albahrani, Hassan A Alramadan, Qasem A Albahrani

**Affiliations:** 1 Pediatrics, Maternity and Children Hospital, Al-Mubarraz, SAU; 2 Neonatology, Maternity and Children Hospital, Al-Mubarraz, SAU; 3 Pediatrics, Johns Hopkins Aramco Healthcare, Dhahran, SAU; 4 Pediatrics, Al Jafr General Hospital, Al-Jafr, SAU; 5 Pediatrics, King Faisal University, Al-Hofuf, SAU

**Keywords:** al ahsa, saudi arabia, risk factor, neonatal intensive care unit, neonate, bleeding, thrombocytopenia

## Abstract

Background

Thrombocytopenia is the most prevalent hematological condition in neonates that develops in the neonatal intensive care unit (NICU). This set of illnesses is caused by either decreased platelet production due to placental insufficiency, increased platelet breakdown (consumption), or a combination of the two causes. Based on platelet count, it is defined as mild, moderate, or severe thrombocytopenia, with early and late onset.

Purpose

The purpose of this study is to determine the prevalence of thrombocytopenia and the factors that contribute to it in newborns hospitalized in the neonatal critical care unit at the Maternity and Children Hospital in Al Ahsa, Saudi Arabia.

Methods

This descriptive retrospective cross-sectional study was carried out at the NICU of the Maternity and Children Hospital in Al Ahsa, Saudi Arabia, over the span of one year (August 2022 to August 2023) among hospitalized neonates with thrombocytopenia. Thrombocytopenia is defined as a platelet count of 150,000 or less. These patients were monitored until they recovered or died.

Results

The inclusion criteria were met by a total of 242 newborns with thrombocytopenia. Half of the neonates (57%) were full-term, with Apgar scores greater than 5 at the first (84%) and fifth (93%) minutes, respectively. The great majority of individuals (84%) experienced early-onset thrombocytopenia of mild severity (62%) and were asymptomatic (93%). The majority of the cases resolved spontaneously, with only 21% requiring platelet transfusion. There was a significant relationship discovered between gestational age and the severity of thrombocytopenia, with very preterm infants having moderate to severe thrombocytopenia, as well as birth weight (p=0.001). Furthermore, neonates with severe thrombocytopenia had a considerably higher mortality rate (p=0.001).

Conclusion

The mortality and morbidity of newborns with perinatal risk for neonatal thrombocytopenia can be reduced with timely detection of the cause and development of thrombocytopenia, as well as adequate and early care.

## Introduction

Platelet production in the fetus begins around the fifth week of gestation. The fetus has a platelet count in the typical range of 150 to 450 109/L by the end of the second trimester [1,2]. Thrombocytopenia can occur throughout pregnancy, and neonates have a low platelet count at delivery. Thrombocytopenia is a prevalent clinical condition among newborns admitted to neonatal intensive care units (NICUs) around the world, and it is analogous to other hematologic disorders of newborns. The prevalence of thrombocytopenia in newborns varies greatly depending on the demographic investigated. When compared to neonates hospitalized in intensive care facilities, the prevalence of neonatal thrombocytopenia (NTP) ranges from 1% to 5% of all newborns. Thrombocytopenia develops in 22-35% of all hospitalizations, with the risk increasing as gestational age decreases [3]. Thrombocytopenia is defined as a platelet count of less than 150,000 platelets per microliter of blood, regardless of gestational age, and is classified based on platelet count as mild thrombocytopenia with a platelet count of 100 to 149 103/L, moderate thrombocytopenia with a platelet count of 50 to 99 103/L, and severe thrombocytopenia with a platelet count of less than 50 x 103/L. It is further classified according to the time of occurrence, with early-onset thrombocytopenia occurring before 72 hours after birth and late-onset thrombocytopenia occurring beyond 72 hours after birth [4,5]. The causes of NTP can be divided into maternal, perinatal, and neonatal causes according to the timing of the condition's start (early vs. late), gestational age (term vs. preterm), the underlying mechanism (increased destruction (consumption) of platelets, or a mix of both processes), and the general health of the newborn [6,7]. The best way to categorize NTP causes into fetal, early-onset, and late-onset is by when they first manifest. Early-onset NTP (72 hours of life) has been linked to placenta insufficiency, hypoxic-ischemic encephalopathy, perinatal or congenital infections, neonatal alloimmune thrombocytopenia, and maternal idiopathic thrombocytopenic purpura. On the other hand, necrotizing enterocolitis (NEC) and late-onset sepsis are linked in the majority of late-onset NTP cases (>72 hours of life) [8].

The hemostasis was greatly impacted by NTP. It can result in cerebral hemorrhage, gastrointestinal bleeding, and pulmonary bleeding. Intracranial hemorrhage (ICH), which affects up to 25% of infants with low birth weight, is more likely to occur in preterm neonates [9]. ICH, which is found in 10-20% of affected fetuses/neonates and often develops within a week of life, is the most dangerous complication of severe fetal and NTP. Twenty percent of ICHs result in neurological squeals, and 5-10% of them result in fatalities [10]. Thrombocytopenia has been documented in up to 75% and 90% of preterm newborns with birth weights less than 1000g and 750g, respectively. According to an expert analysis of a hematology report, low-birthweight infants have a 2.5fold greater risk of thrombocytopenia [11].

However, because there is little local research on the incidence of NTP and related risk factors in Saudi Arabia, the purpose of this study is to ascertain the prevalence of NTP, identify risk factors, and assess the results.

## Materials and methods

Aim of the study

This study aims to determine the prevalence of neonatal thrombocytopenia and its associated risk factors in the NICU of the Maternity and Children Hospital in Al Ahsa, Saudi Arabia.

Study design and population

Institution-based descriptive retrospective research was undertaken over a 12-month period (August 2022 to August 2023) on neonates admitted to the NICU at the maternity and pediatrics hospital in Al Ahsa, Saudi Arabia. All newborns admitted to the NICU throughout the research period had their medical records reviewed retrospectively. A simple sampling strategy was used to enroll neonates with a platelet count of 150,000 upon NICU admission in the trial.

Inclusion and exclusion criteria

Any neonate patient (<28 days) admitted in the NICU of the Maternity and Children Hospital in Al Ahsa, Saudi Arabia, with thrombocytopenia <150,000 that was confirmed by at least two readings was included. The pediatric age group (>28 days) who were not admitted to the NICU with a normal platelet count of ≥150,000 or had thrombocytopenia from a single reading were excluded.

Data collection

Gender, gestational age at delivery, birth weight, clinical diagnosis of NTP, platelet count, treatment methods, results, and mother features, as well as maternal obstetric difficulties, pre-existing maternal chronic conditions, and obstetric history during pregnancy, were all extracted from the medical records of eligible patients. Preterm birth is defined as giving birth before 37 weeks of pregnancy. It is further divided into four categories: extreme preterm (less than 28 weeks), very preterm (between 28 and 32 weeks), moderate preterm (between 32 and 34 weeks), and late preterm (between 34 and 37 weeks). The neonates were categorized according to their birth weight into four categories: normal birth weight (2500 g), low birth weight (2500 g), very low birth weight (1500 g), and extremely low birth weight (1000 g). Early (appearing within the first 72 hours of life) or late (occurring after 72 hours) onset patterns of NTP were distinguished. The severity of NTP was divided into three categories based on platelet counts: mild (platelet counts between 100 and 150 109/L), moderate (platelet counts between 50 and 99 109/L), and severe (platelet counts below 50 109/L).

Data analysis

All data is statistically processed and displayed using tables and images. The standard methods of descriptive statistics were used in the analysis. Absolute numbers and percentages are used to describe categorical data. The mean value ± standard deviation, or median, is used to describe numerical data. Statistical data analysis was carried out using SPSS Statistics version 22 (IBM Corp. Released 2013. IBM SPSS Statistics for Windows, Version 22.0. Armonk, NY: IBM Corp.). Bivariate and multivariable logistic regression were carried out to see associations between dependent and independent variables. Those variables that have a p-value <0.05 in bivariable logistic regression were taken to the multivariable logistic regression model.

## Results

A total of 242 neonates with thrombocytopenia were included. About 140 (57.9%) were males, and the vast majority (213, 88%) were Saudi. Exact of 22 (9.1%) had extreme preterm, 27 (11.2%) were very preterm, and 139 (57.4%) were full-term. As for birth weight, 27 (11.2%) had very extremely low BW, 25 (10.3%) had very low BW, and 118 (48.8%) had normal BW. Normal vaginal delivery (NVD) was reported among 129 (53.3%), while 98 (40.5%) needed an emergent cesarean section (CS). Exact of 37 (15.3%) had an APGAR score at the first minute less than 5, 16 (6.8%) had a score less than 5 after five minutes, and only seven (18.9%) after 10 minutes. The most reported blood groups were O+ (47.9%), A+ (21.9%), B+ (21.9%), and AB+ (3.3%) (Table 1).

**Table 1:** Bio-demographic data of neonates with thrombocytopenia in the NICU (n=242) PT: preterm, ELBW: extremely low birth weight, VLBW: very low birth weight, LBW: low birth weight, NBW: normal birth weight, NVD: normal vaginal delivery, CS: cesarean section, NICU: neonatal intensive care unit

Bio-demographic data	No	%
Gender		
Male	140	57.9%
Female	102	42.1%
Nationality		
Saudi	213	88.0%
Non-Saudi	29	12.0%
Gestational age (weeks)		
Extreme PT (<28 W)	22	9.1%
Very PT (28 to <32 W)	27	11.2%
Moderate PT (32 to <34 W)	12	5.0%
Late PT (34 to <37 W)	42	17.4%
Full term (37 to 42 W)	139	57.4%
Birth weight (grams)		
ELBW (<1000 g)	27	11.2%
VLBW (1000 to 1499 g)	25	10.3%
LBW (1500 to 2499 g)	72	29.8%
NBW (2500 to 3999 g)	118	48.8%
Mode of delivery		
NVD	129	53.3%
Assisted vaginal delivery	5	2.1%
Elective CS	98	40.5%
Emergency CS	10	4.1%
APGAR score at one minute		
<5	37	15.3%
≥5	205	84.7%
Mean ± SD	6.7 ± 2.1	
APGAR score at five minutes		
<5	16	6.8%
≥5	218	93.2%
Mean ± SD	7.3 ± 1.4	
APGAR score at 10 minutes		
<5	7	18.9%
≥5	30	81.1%
Mean ± SD	5.8 ± 1.8	
Blood group		
O+	116	47.9%
A+	53	21.9%
B+	53	21.9%
AB+	8	3.3%
O-	6	2.5%
A-	3	1.2%
B-	3	1.2%

Clinical data on thrombocytopenia among neonates in the NICU. A total of 205 (84.7%) of the neonates showed thrombocytopenia within the first 72 hours. Thrombocytopenia was mild among most of the neonates (152, 62.8%), moderate among 55 (22.7%), and severe among 35 (14.5%). The vast majority of the neonates had asymptomatic thrombocytopenia (225, 93%), while among the symptomatic, skin manifestation was the most reported among nine (42.9%), ICH among seven (33.3%), and pulmonary hemorrhage among four (19%). DCT was positive among 22 (9.1%) neonates. Regarding treatment, most of the cases needed observation only (169, 69.8%), while platelet transfusion was needed among 51 (21.1%) cases, which was for one time among 28 (52.8%), for two to three times among 15 (28.3%), and for more than three times among 10 (18.9%). Intravenous immunoglobulin was used among 15 (6.2%), which was one time among 12 (92.3%). Only seven (2.9%) cases had steroids (more than three times) (Table 2).

**Table 2:** Clinical data of thrombocytopenia among neonates in NICU ICH: intracerebral hemorrhage, DCT: direct Coombs test, IVGI: intravenous immunoglobulin, NICU: neonatal intensive care unit

Clinical data	No	%
Thrombocytopenia onset		
≤72 hours (early)	205	84.7%
>72 hours (late)	37	15.3%
Thrombocytopenia severity		
Mild (149,000-100,000)	152	62.8%
Moderate (99,000-50,000)	55	22.7%
Severe (less than 50,000)	35	14.5%
Thrombocytopenia presentation		
Asymptomatic	225	93.0%
Symptomatic	17	7.0%
Symptoms		
Skin manifestation	9	42.9%
ICH	7	33.3%
Pulmonary hemorrhage	4	19.0%
Gastrointestinal hemorrhage	1	4.8%
DCT		
Negative	220	90.9%
Positive	22	9.1%
Treatment received		
Observation only	169	69.8%
Platelets transfusion	51	21.1%
Fresh frozen plasma	42	17.4%
Packed red blood cells	37	15.3 %
Intravenous immunoglobulin	15	6.2%
Steroids	7	2.9%
If platelets are transfused, how many time		
1 time	28	52.8%
2-3 times	15	28.3%
>3 times	10	18.9%
If steroids are given, how many time		
>3 times	7	100.0%
If IVGI is given, how many times		
1 time	12	92.3%
2-3 times	1	7.7%

**Figure 1:**
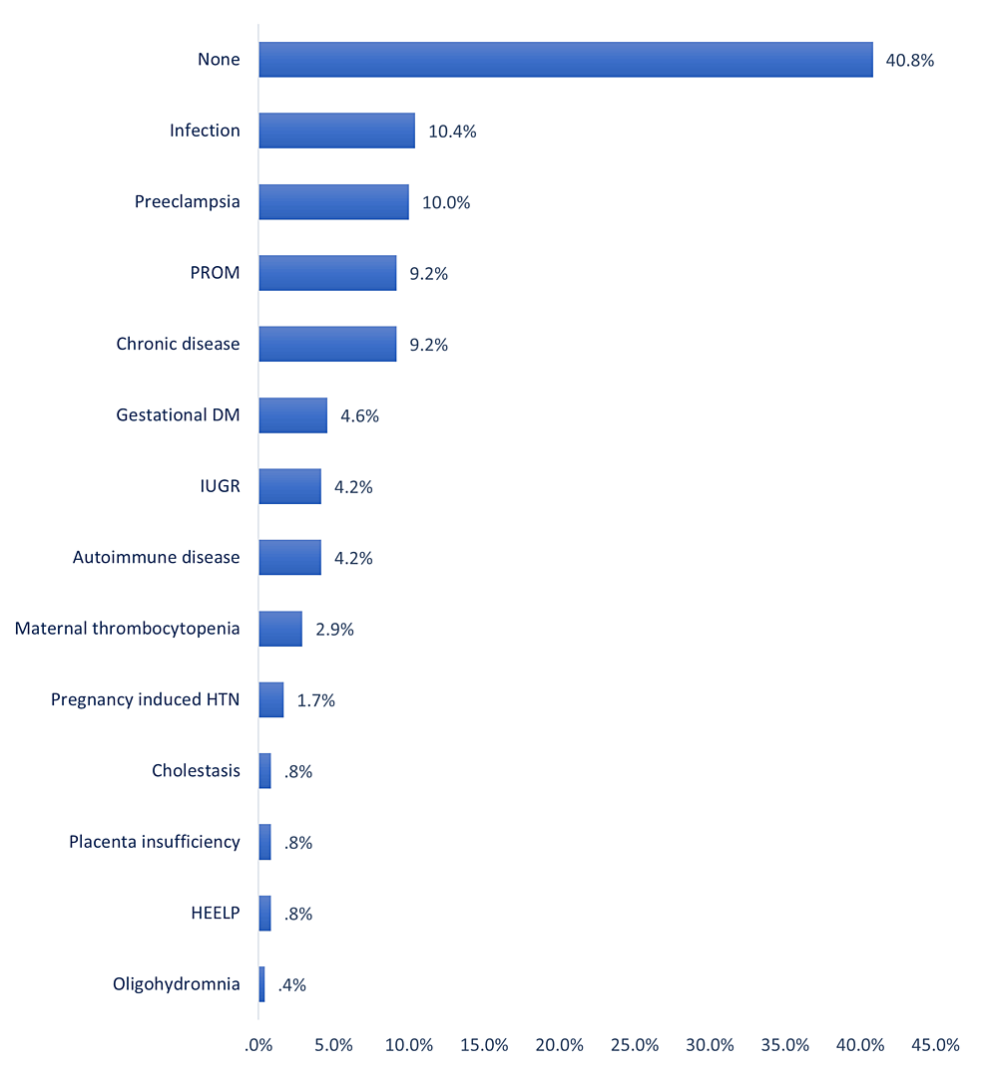
Antenatal risk factors of thrombocytopenia among neonates in NICU PROM: premature rupture of membranes, DM: diabetes mellitus, HTN: hypertension, HELLP: hemolysis, elevated liver enzymes, and low platelet syndrome, IUGR: intrauterine growth restriction, NICU: neonatal intensive care unit

Antenatal risk factors for thrombocytopenia among neonates in a NICU in Al-Ahsa, Saudi Arabia. The most reported antenatal risk factors (maternal) were infection (10.4%), pre-eclampsia (10%), premature rupture of membranes (PROM; 9.2%), having chronic disease (9.2%), gestational diabetes mellitus (DM; 4.6%), intrauterine growth restriction (IUGR; 4.2%), having autoimmune disease (4.2%), and maternal thrombocytopenia (2.9%). Exactly 40.8% of the neonates had no antenatal risk factors (Figure 1).

**Figure 2:**
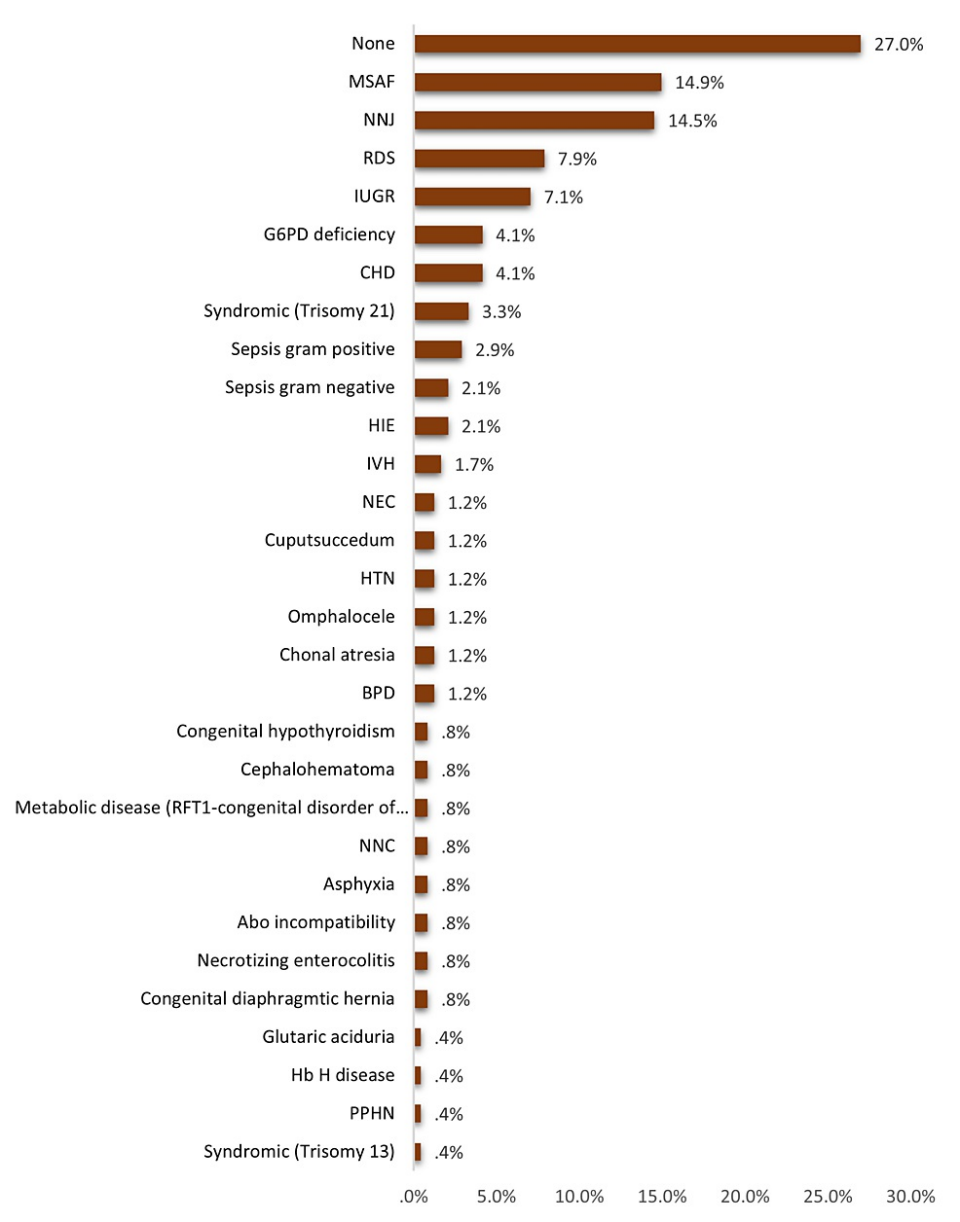
Postnatal risk factors of thrombocytopenia among neonates in NICU MSAF: meconium-stained amniotic fluid, NNJ: neonatal jaundice, RDS: respiratory distress syndrome, IUGR: intrauterine growth restriction, G6PD: glucose-6-phosphate dehydrogenase, CHD: congenital heart disease, HIE: hypoxic-ischemic encephalopathy, IVH: intraventricular hemorrhage, NEC: necrotizing enterocolitis, HTN: hypertension, BPD: bronchopulmonary dysplasia, NNC: neonatal convulsion, PPHN: persistent pulmonary hypertension of the newborn, NICU: neonatal intensive care unit

Postnatal risk factors (neonatal) for thrombocytopenia among neonates in the NICU. The most reported postnatal risk factors included meconium-stained amniotic fluid (MSAF; 14.9%), neonatal jaundice (NNJ; 14.5%), respiratory distress syndrome (RDS; 7.9%), IUGR (7.1%), glucose-6-phosphate dehydrogenase (G6PD) deficiency (4.1%), congenital heart disease (CHD; 4.1%), and trisomy 21 (3.3%). A total of 27% of the cases had no postnatal risk factors (Figure 2).

**Figure 3:**
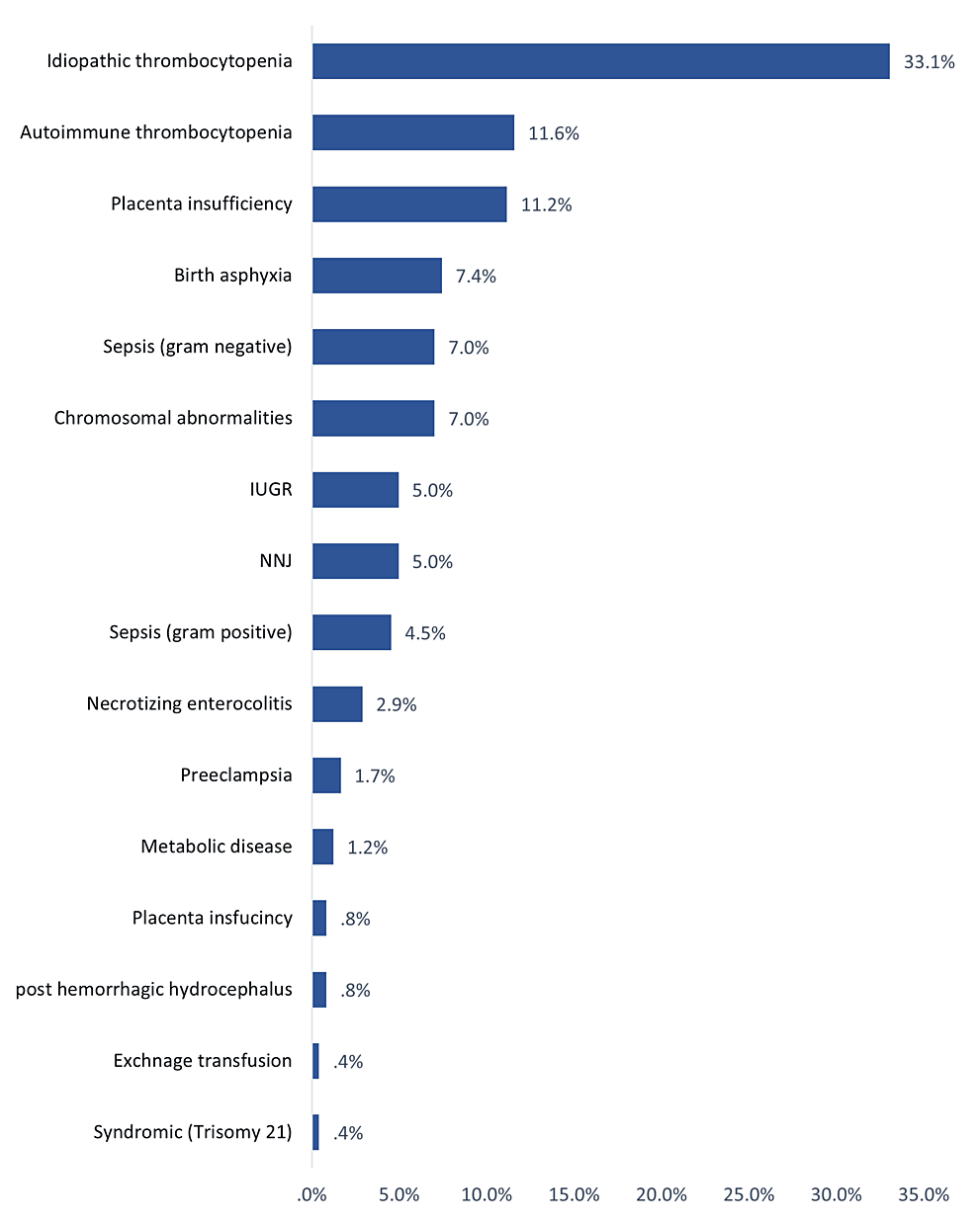
Most likely cause of this thrombocytopenia among study neonates in NICU NNJ: neonatal jaundice, IUGR: intrauterine growth restriction, NICU: neonatal intensive care unit

The most likely cause of this thrombocytopenia among study neonates in the NICU. The most likely causes were idiopathic (33.1%), autoimmune thrombocytopenia (11.6%), placental insufficiency (11.2%), birth asphyxia (7.4%), sepsis by a gram-negative organism (7%), and chromosomal anomalies (7%) (Figure 3).

Outcome among neonates with thrombocytopenia in the NICU. A total of 86 (35.5%) of the neonates had critical cases. Exactly 123 (50.8%) resolved within 48 hours, 50 (20.7%) resolved within 48 hours, and 40 (16.5%) resolved after five days. The in-hospital mortality rate was 12% (Table 3).

**Table 3:** Outcome among neonates with thrombocytopenia in NICU NICU: neonatal intensive care unit

Outcome	No	%
Clinical condition		
Stable	156	64.5%
Critical	86	35.5%
Clinical outcome		
Resolved within 48 hours	123	50.8%
Resolved 3-5 days	50	20.7%
Resolved >5 days	40	16.5%
Mortality	29	12.0%

Factors associated with thrombocytopenia severity among neonates in the NICU. Exact of 70.5% of full-term neonates had mild thrombocytopenia versus 40.9% of others with extreme preterm, with recorded statistical significance (p=0.013). Also, 74.6% of full-term neonates had mild thrombocytopenia compared to 22.2% of those with extremely low birth weight (p=0.001). Mild thrombocytopenia was detected in 80% of neonates with no postnatal risk factors, compared to 56.5% of those with (p=0.004). Likewise, 81.3% of neonates had idiopathic thrombocytopenia versus 53.7% of those who had secondary thrombocytopenia (p=0.001) (Table 4).

**Table 4:** Factors associated with thrombocytopenia severity among neonates in NICU P: Pearson X2 test, $: exact probability test, * p<0.05 (significant) PT: preterm, ELBW: extremely low birth weight, VLBW: very low birth weight, LBW: low birth weight, NBW: normal birth weight, NVD: normal vaginal delivery, CS: cesarean section, NICU: neonatal intensive care unit

Factors	Thrombocytopenia severity						p-value
	Mild		Moderate		Severe		
	No	%	No	%	No	%	
Gender							0.326
Male	92	65.7%	27	19.3%	21	15.0%	
Female	60	58.8%	28	27.5%	14	13.7%	
Gestational age (weeks)							0.013*^$^
Extreme PT (<28 W)	9	40.9%	10	45.5%	3	13.6%	
Very PT (28 to <32 W)	15	55.6%	5	18.5%	7	25.9%	
Moderate PT (32 to <34 W)	8	66.7%	2	16.7%	2	16.7%	
Late PT (34 to <37 W)	22	52.4%	9	21.4%	11	26.2%	
Full term (37 to 42 W)	98	70.5%	29	20.9%	12	8.6%	
Birth weight (grams)							0.001*
ELBW (<1000 g)	6	22.2%	13	48.1%	8	29.6%	
VLBW (1000 to 1499 g)	20	80.0%	2	8.0%	3	12.0%	
LBW (1500 to 2499 g)	38	52.8%	20	27.8%	14	19.4%	
NBW (2500 to 3999 g)	88	74.6%	20	16.9%	10	8.5%	
Mode of delivery							0.119$
NVD	89	69.0%	26	20.2%	14	10.9%	
Assisted vaginal delivery	2	40.0%	3	60.0%	0	0.0%	
Elective CS	56	57.1%	24	24.5%	18	18.4%	
Emergency CS	5	50.0%	2	20.0%	3	30.0%	
Antenatal risk factors							0.799
No	64	65.3%	21	21.4%	13	13.3%	
Yes	88	61.1%	34	23.6%	22	15.3%	
Postnatal risk factors (neonatal)							0.004*
No	52	80.0%	8	12.3%	5	7.7%	
Yes	100	56.5%	47	26.6%	30	16.9%	
The most likely cause of this thrombocytopenia							0.001*
Idiopathic	65	81.3%	9	11.3%	6	7.5%	
Secondary	87	53.7%	46	28.4%	29	17.9%	

Relationship between thrombocytopenia severity and clinical outcome among neonates in the NICU. A total of 71.1% of neonates with mild thrombocytopenia had stable conditions versus 54.3% of others with severe thrombocytopenia (p=0.021). As for mortality rate, it was 5.3% among cases with mild thrombocytopenia compared to 21.8% of those with mild thrombocytopenia and 25.7% of cases with severe thrombocytopenia (p=0.001) (Table 5).

**Table 5:** Relationship between thrombocytopenia severity clinical outcome among neonates in NICU P: Pearson X2 test, * p<0.05 (significant) NICU: neonatal intensive care unit

Outcome	Thrombocytopenia severity						p-value
	Mild		Moderate		Severe		
	No	%	No	%	No	%	
Clinical condition							0.021*
Stable	108	71.1%	29	52.7%	19	54.3%	
Critical	44	28.9%	26	47.3%	16	45.7%	
Clinical outcome							0.001*
Resolved within 48 hours	97	63.8%	21	38.2%	5	14.3%	
Resolved 3-5 days	28	18.4%	11	20.0%	11	31.4%	
Resolved >5 days	19	12.5%	11	20.0%	10	28.6%	
Mortality	8	5.3%	12	21.8%	9	25.7%	

## Discussion

Thrombocytopenia is the most frequent hematological issue found in newborns, excluding anemia caused by phlebotomy [12,13]. It is particularly common in NICUs, which might lead some to dismiss it as a non-issue and simply administer platelet transfusions when the count drops below a certain level [14-16]. However, there are numerous reasons why this approach should be reconsidered. Researchers are gaining greater insight into the underlying causes of NTP, and many commonly held beliefs are being debunked due to a lack of evidence [17]. By identifying the specific causes and mechanisms of this condition, clinicians can develop more effective treatments, including innovative approaches.

The current study aimed to assess the prevalence and associated risk factors for NTP among newborns admitted to the NICU. The study showed that more than half of the cases were male and full-term, and about half of them had a normal birth weight. NVD was the dominant mode of delivery, but elective CS was reported in less than half of the cases. Positive blood groups, mainly blood group O, were the most frequent. Similar findings were reported by Wodaje et al. [18], where 54.1% of neonates with thrombocytopenia were males, 49.9% were born through NVD, 57.4% had a normal birth weight, and 52.7% were full-term. Also, Abebe Gebreselassie et al. [19] documented that 56.2% were males and 70% had a normal birth weight.

As for thrombocytopenia clinical data, the current study showed that most of the neonates showed thrombocytopenia within the first 72 hours. Thrombocytopenia was mild among about two-thirds of the neonates, moderate among about one-fifth, and severe among 14.5%. The vast majority of the neonates had asymptomatic thrombocytopenia, while among the symptomatic, skin manifestation was the most reported, followed by ICH and pulmonary hemorrhage. Similar findings were reported in the literature, as most of the neonates (>75%) developed early-onset thrombocytopenia due to placental insufficiency/fetal hypoxia [20,21]. In Saudi Arabia, a similar conclusion was documented by Eltawel et al. [22], as the average time to disease onset was 1.83 days, whereas that of recovery duration was 15.35 (18.46) days. In contrast, Wodaje et al. [18] found that 26.2% had mild thrombocytopenia, 38.3% had moderate thrombocytopenia, and 35.5% had severe thrombocytopenia. As for the onset of thrombocytopenia, 28% had early-onset thrombocytopenia and 72% had late-onset thrombocytopenia. Also, Murray et al. [23] found that thrombocytopenia developing, or clinically worsening, after 72 hours is practically fully caused by late-onset sepsis or NEC.

As for risk factors, the current study assessed prenatal and postnatal risk factors. The most reported antenatal risk factors (maternal) were infection, pre-eclampsia, PROM, chronic disease, gestational DM, IUGR (4.2%), and autoimmune disease. Most of the neonates had no antenatal risk factors. Considering postnatal risk factors, the most reported included MASF, NNJ, RDS, IUGR, G6PD, CHD, and Trisomy 21. About one-fourth of the cases had no postnatal risk factors. The most likely causes were being idiopathic (about one-third of the cases, while autoimmune thrombocytopenia, placental insufficiency, birth asphyxia, and sepsis were the most frequent secondary probable causes. Sepsis with the presence of sepsis and atresia were the significantly associated factors with the development of thrombocytopenia reported by Abebe Gebreselassie et al. [19]. Sepsis was also the dominant risk factor for NTP among many other studies from Nigeria, Turkey, India, Iran, Indonesia, and Austria [24-29]. Other risk factors were reported by Wodaje et al. [18] including eclampsia, prolonged rupture of membrane, intrauterine growth retardation, perinatal asphyxia, and NEC. The association between thrombocytopenia incidence and similar risk factors such as intrauterine growth retardation, asphyxia, gestational DM, maternal hypertension, and prematurity in other many literature studies [14,30,31]. Regarding factors associated with the severity of thrombocytopenia, the current study showed pre-term (p=0.013), low birth weight (p=0.001), and postnatal risk factors and secondary thrombocytopenia (p=0.001) were the significant reported factors.

Regarding treatment, more than two-thirds needed observation only, while platelet transfusion was needed for about one-fifth of the study cases, which was for one time among about half of them and two to three times among more than one-fourth.

With regard to clinical outcomes, about one-third of the neonates had critical cases. About half of them were resolved within 48 hours. The in-hospital mortality rate was 12%. Higher mortality was associated with the severity of thrombocytopenia. A similar mortality rate (12.5%) was reported by Resch et al. [29], but a higher mortality rate (20.2%) was reported by Zekry et al. [32].

Limitation

This research study provides valuable insights into the prevalence, risk factors, and outcomes associated with thrombocytopenia among neonates, although it has certain limitations. One of the limitations of this study is its generalizability. It should be conducted among a larger sample size and include participants from different regions of Saudi Arabia. More specific information about several variables could not be obtained due to the retrospective methodology used in this study. Moreover, patients with incomplete medical records were excluded from the ongoing study. Lastly, the guidelines for platelet transfusion in neonates were available but were not strictly followed while transfusing platelets in the NICU. The current study can provide baseline data for future prospective, multi-center research.

## Conclusions

The current study showed that most NTP was among males with normal birth weight and full-term. Most cases had early-onset thrombocytopenia, mainly asymptomatic, which was idiopathic, with some reported prenatal and postnatal risk factors, mainly infections and sepsis. Also, most cases were stable, with half of them resolved within 48 hours and a low in-hospital mortality rate. It is important to consider the risk factors for life-threatening events in newborns with thrombocytopenia to prevent future complications.
